# Quantitative spatial mapping of distorted state phases during the metal-insulator phase transition for nanoscale VO_2_ engineering

**DOI:** 10.1080/14686996.2022.2150525

**Published:** 2022-12-23

**Authors:** Yuichi Ashida, Takafumi Ishibe, Jinfeng Yang, Nobuyasu Naruse, Yoshiaki Nakamura

**Affiliations:** a Graduate School of Engineering and Science, Osaka University, Toyonaka, Japan; b The Institute of Scientific and Industrial Research, Osaka University, Ibaraki, Japan; c Department of Fundamental Bioscience, Shiga University of Medical Science, Otsu, Japan

**Keywords:** VO_2_ strained states, phase transition control, geometric phase analysis, VO_2_ nanostructure

## Abstract

Vanadium dioxide (VO_2_) material, known for changing physical properties due to metal-insulator transition (MIT) near room temperature, has been reported to undergo a phase change depending on the strain. This fact can be a significant problem for nanoscale devices in VO_2_, where the strain field covers a large area fraction, spatially non-uniform, and the amount of strain can vary during the MIT process. Direct measurement of the strain field distribution during MIT is expected to establish a methodology for material phase identification. We have demonstrated the effectiveness of geometric phase analysis (GPA), high-resolution transmission electron microscopy techniques, and transmission electron diffraction (TED). The GPA images show that the nanoregions of interest are under tensile strain conditions of less than 0.4% as well as a compressive strain of about 0.7% (Rutile phase VO_2_[100] direction), indicating that the origin of the newly emerged TED spots in MIT contains a triclinic phase. This study provides a substantial understanding of the strain-temperature phase diagram and strain engineering strategies for effective phase management of nanoscale VO_2_.

## GRAPHICAL ABSTRACT

**Figure:**
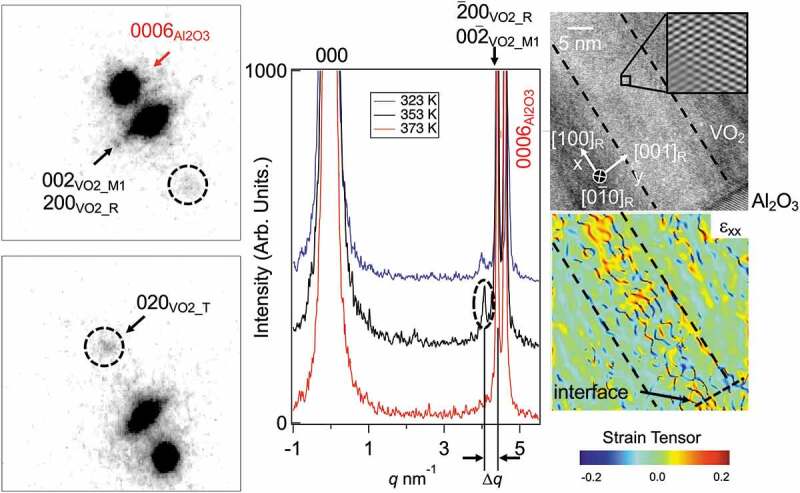

## 1. Introduction

Vanadium dioxide (VO_2_) exhibits a metal-insulator transition (MIT) due to external fields such as heat [1,2], light [3,4], electric fields [5,6], and magnetic fields [7], which are accompanied by structural phase transition. In addition, strain [2,8,9], and doping [10–15] change the MIT conditions and properties. Since the electrical and optical properties of VO_2_ change significantly with the phase transition, applications in photonics and electronics are particularly promising [16–21]. Some optical switches have been developed combining VO_2_ exhibiting the MIT with a silicon waveguide for miniaturizing optical switches [7]. The low-temperature VO_2_ phases include not only the well-known M1 phase (monoclinic) but also two metastable structures, the insulating M2 phase (monoclinic) and T phase (triclinic), due to stoichiometric defects and strain effects, which can pose challenges for nanoscale devices [22,23]. Due to their metastable structures and spatial phase inhomogeneities in the film/bulk samples, these structural phases can exhibit different phase transition behaviors and properties. In recent years, a wide range of device applications involving miniaturization using nanostructures and nanofilms of oxides have been expanding [24–26]. Precise control of domain structures and phase transitions at the single domain level is also expected for VO_2_ materials, and the development of a method to precisely characterize the phase structure of VO_2_ at the nanoscale is a major challenge [27].

According to Wang *et al*. [28] and Kim *et al*. [29], the M2 and T phases are stabilized by doping, external strain, and oxygen nonstoichiometry. Effective strain control strategies are necessary to create VO_2_ crystals with stabilized multiphase because doping and stoichiometry can cause large structural distortions in the VO_2_ lattice, changing its intrinsic properties and applications. Strained VO_2_ fields are not a problem in conventional macroscopic devices but are critical in nanoscale devices; the area covered by the strained field is large and spatially non-uniform, and the amount of strain can change during the phase transition process. However, the challenge is that there is no way to quantitatively measure the strain state of those VO_2_ in actual nanomaterials.

In this study, using geometric phase analysis (GPA), an analysis of high-resolution electron microscopy (HRTEM), we have exhibited crystallographic evidence from transmission electron diffraction (TED) that there is indeed an intermediate phase of the MIT while quantitatively evaluating the strain in thin films of VO_2_ grown on sapphire. In order to precisely control VO_2_ and even the properties of the phase transition process in the future, we need a recipe to quantitatively measure the strain of the desired nanostructure. We will demonstrate that GPA and TED can be used to quantitatively evaluate the strain of phase transitions in the single domain, which was not readily apparent due to the sub-resolution displacement of HRTEM.

## 2. Experimental details

A c-cut sapphire substrate (10 × 10 mm^2^) was ultrasonically cleaned with acetone, ethanol, and an ultrapure water bath for 5 min each. After removing the surface moisture with a nitrogen gun, the washed sample was introduced into a load lock chamber. Then, the sample was transferred to a deposition chamber equipped with a pulse laser deposition system (Lambda Physics) [25]. A V_2_O_5_ target was used for the growth of VO_2_ film. The deposition chamber was evacuated to a   2 × 10^−5^ Pa prior to deposition. The thickness of the VO_2_ thin film of 100 nm was formed on the substrate by pulsed ArF laser deposition (wavelength 248 nm, energy 50 mJ, frequency 10 Hz, substrate temperature 723 K, O_2_ pressure 1.0 Pa). The temperature dependence of the electrical conductivity of the VO_2_ film was measured between 300 and 400 K by the van der Pauw method (self-made) to confirm the phase transition temperature (Supporting information Figure S1(a)). The crystallinity of the fabricated VO_2_ film was evaluated by X-ray diffraction (XRD) measurements (Rigaku, Ultima IV) with 2θ-ω scan (Supporting information Figure S1(b)).

Focused ion beam processing (Thermo Fisher Scientific, Scios 2) was performed to thin the VO_2_ film so that it could be observed from [010]_M1_ (Rutile phase VO_2_[$0\overset{ˉ}{1}0$]_R_) orientation. The TED pattern and cross-sectional HRTEM were taken at 200 keV (HITACHI, HF-2000). The temperature was repeatedly lowered and raised 323–373 K (323 K, 353 K, 373 K) in the TEM with a temperature variable holder (Gatan, 628). The temperature was held for about 15 minutes until each temperature became constant, and then HRTEM and TED observations were performed. There was no significant change in the diffraction pattern at the same temperature when the temperature was raised and lowered. Geometric Phase Analysis (GPA) was used to visualize the strain changes in the VO_2_ film using Grillo’s algorithm STEM_cell [30]. The diffracted spots of VO_2_ (002)_M1_ and ($20\overset{ˉ}{2}$)_M1_ were used as reference points for the GPA. The output GPA images are 16 bits.

## 3. Results and discussion

**Figure 1.**
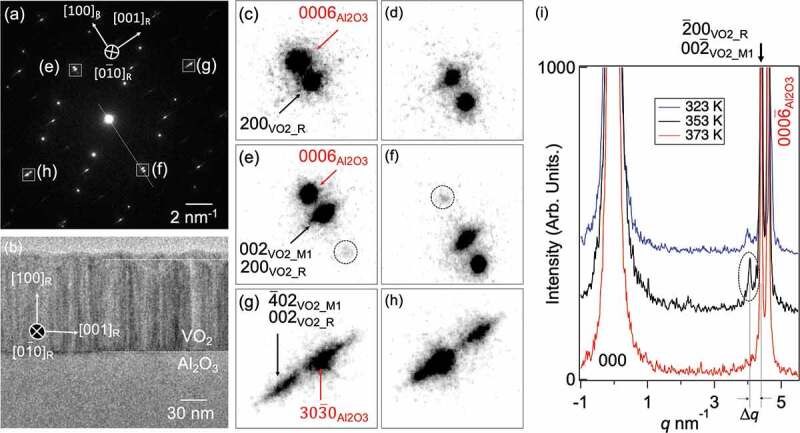
(a) TED pattern viewed from VO_2_[$0\overset{ˉ}{1}0$]_R_ and Al_2_O_3_[$11\overset{ˉ}{2}0$] direction at 353 K. (b) A cross-sectional TEM image for VO_2_ film on Al_2_O_3_ substrate. The magnified image of the SAED around (c) the spot of 200_VO2_R_ and (d) $\overset{ˉ}{2}$00_VO2_R_ at 373 K. (e) and (f) Diffraction spots at 353 K for the same spots as (c) and (d). The diffraction spots surrounded by a circular dotted line appeared only at 353 K. (g) and (h) Magnified image around $\overset{ˉ}{4}$02_VO2_M1_ (002_VO2_R_) and 30$\overset{ˉ}{3}$0_Al2O3_ spots at 353 K. (i) Line profile of the white dotted line in (a) at the temperature of 353 K. The distance Δ*q* between the $\overset{ˉ}{2}$00_VO2_R_ spot and the newly appeared one on the reciprocal space is 0.34 nm^−1^.

The TED pattern shown in Figure 1(a) is taken from VO_2_[010]_M1_ (VO_2_[$0\overset{ˉ}{1}0$]_R_ and Al_2_O_3_[$11\overset{ˉ}{2}0$]) direction, which allows us for detailed measurements of the atomic arrangement changes in M1, M2, T, and R phases. The R phase is known as metallic. The pattern was obtained at 353 K from a 5 μm VO_2_ thin film region together with the sapphire substrate as a reference for d-spacing. As shown in the cross-sectional TEM image (Figure 1(b)) of a VO_2_ film formed on the substrate, VO_2_ domains grow perpendicular to the interface. Although it is difficult to see in the wide-area TEM image in Figure 1(b), the VO_2_ domain is 10–20 nm size in parallel to the interface and is long in the surface direction. Figure 1(c–f) are enlarged images of the area enclosed by the square at 353 K and 373 K. These spots are indexed as 200_VO2_R_ and $\overset{ˉ}{2}$00_VO2_R_. When the sample temperature is further lowered to 353 K form 373 K, the intensity of the newly emerging spots, which could hardly be visualized at 373 K, becomes more intense. The spots appeared along the line parallel to the [100]_R_ direction; it was observed as a strong intensity at 353 K and as a small intensity at 323 K. Streaky diffraction spots were observed parallel to the VO_2_[001]_R_ direction (Figure 1(g,h)). The streak spots correspond to the presence of a VO_2_ single domain with a width of 10–20 nm. The domain size was also confirmed by atomic force microscopy images of the surface (Supporting information Figure S2).

There are two possible explanations for the newly emerging spots. One explanation is that the VO_2_ domain has a T phase with the M1 and R phases due to a strain. The relationship between temperature and strain proposed by Raman spectroscopy has suggested that VO_2_ becomes T phase at 0.5–1% relative strain [31]. Another explanation is the generation of a new long-period strained structure, as discussed later. First, we consider the former in detail. Assuming that the newly emerged spots are the lattice spacing (d-spacing) of the new crystal phase, it corresponds to d = 0.24 ± 0.05 nm. According to the previous reports of XRD, the emerging spots correspond to 020_VO2_T_ on reciprocal space [11,12]. There is no evidence as to how strained the VO_2_ domains are at 353 K, although it has been reported that the area near the interface with sapphire is under a strain state [32]. However, the difference in the arrangement of V atoms between M1, M2, and T phases is reported to be smaller than 0.02 nm, which is difficult to distinguish even with the help of HRTEM image simulations. In addition, the XRD of the T phase was measured by creating a strained state by doping with Al [23], which can be different from the phase caused by external pressure or force fields. Moreover, the biggest question is why the spots that appeared at 353 K were not observed at 323 K.

**Figure 2.**
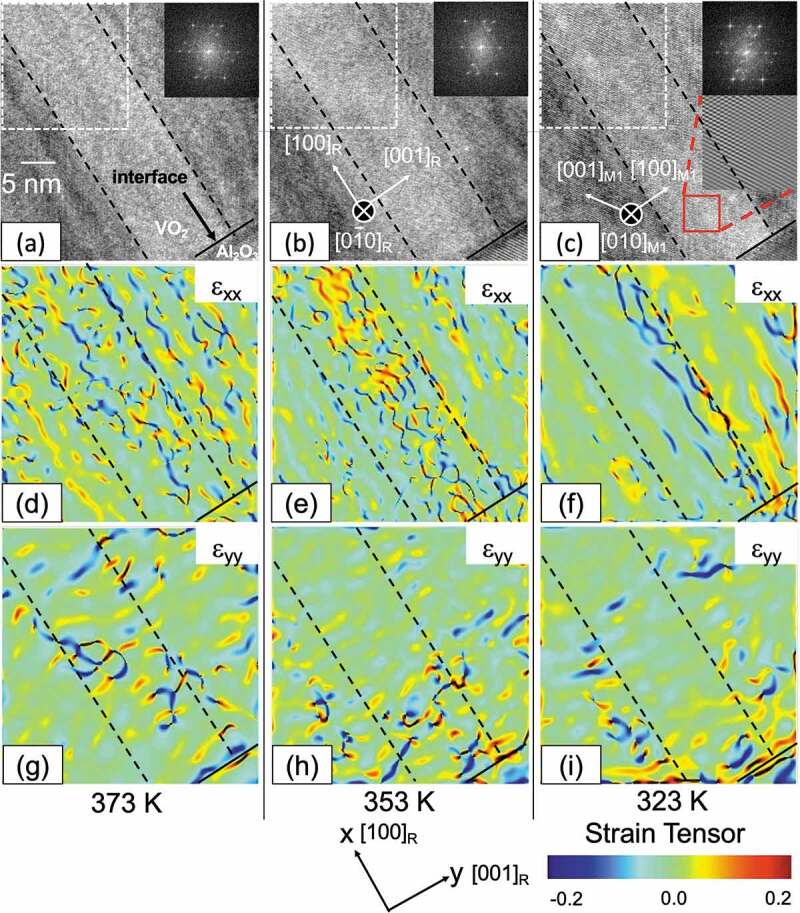
HRTEM images VO_2_ film area together with the interface with Al_2_O_3_ substrate at (a) 373 K, (b) 353 K, and (c) 323 K, respectively. The insets of FFT images were from the area surrounded by the white dashed line. The inset of HRTEM image in (c) shows a magnified image of the red square region, indicating that those HRTEM images are of sufficient quality to perform a geometric phase analysis (GPA) (Supporting information Figure 3S(a–c)). Strain mapping of different strain terms along the (d)–(f) ε_xx_ direction (VO_2_[100]_R_) and (g)–(i) ε_yy_ direction (VO_2_[001]_R_) between (002)_M1_ and (402)_M1_ were obtained by GPA of the HRTEM images at the respective temperature. Black dashed lines in (a)–(i) indicate the VO_2_ domain wall. Solid black lines exhibit the interface between VO_2_ film and Al_2_O_3_ substrate.

**Figure 3.**
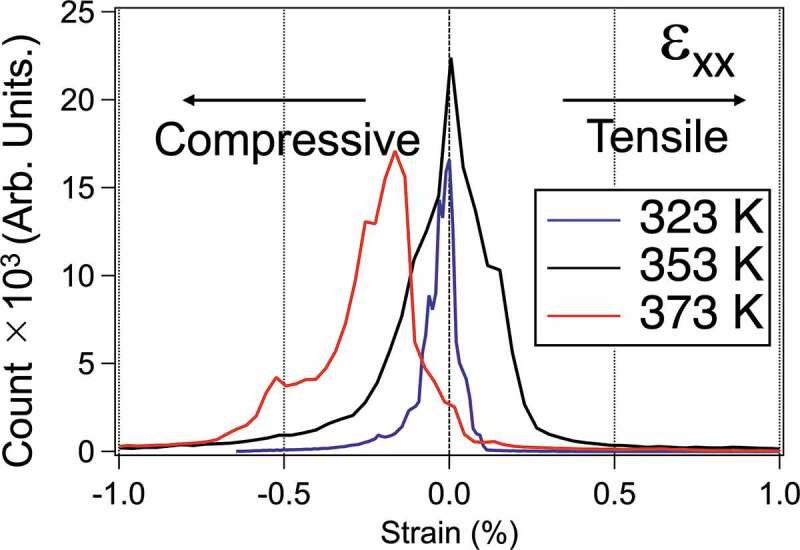
Histograms of strain in the ε_xx_ direction in the area sandwiched by the domain boundary in Figure 2(d–f).

**Figure 4.**
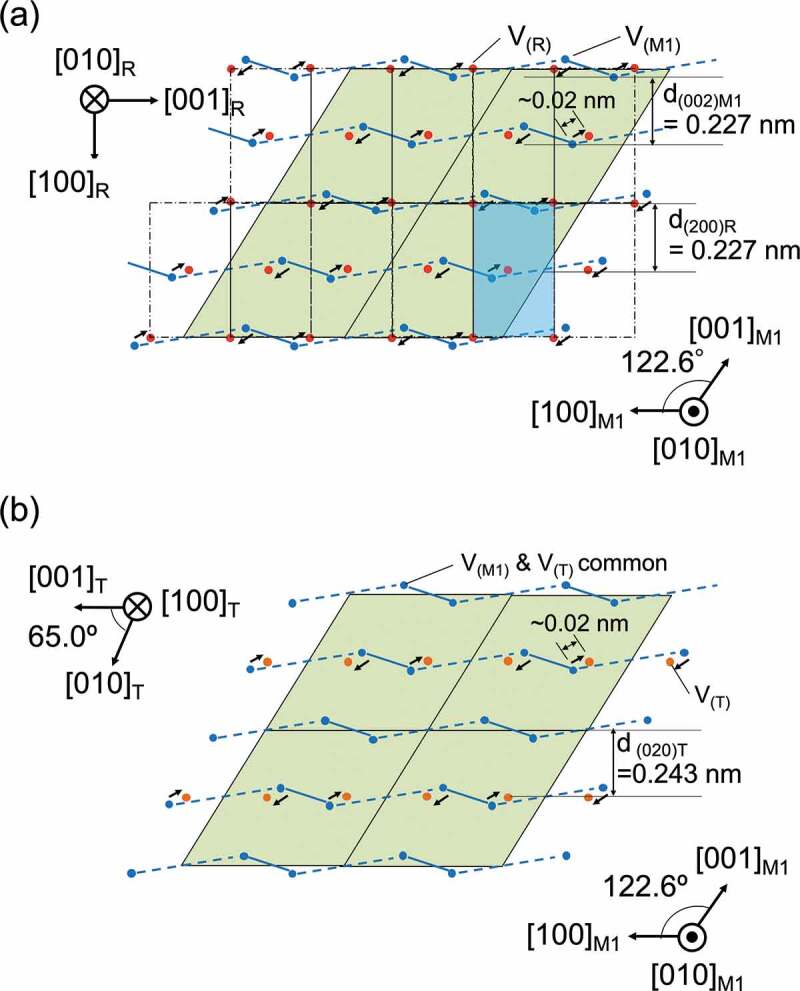
(a) Schematic diagram of V atoms shifts in M1-R phase transition (the spheres indicate positions of the vanadium atoms in the R (red) and M1 (blue) phases). Blue solid lines and dashed blue lines represent the short and long bond lengths. The unit cell for phase R is shown as a blue rectangle, and the unit cell for phase M1 is a green parallelogram. The V-atomic shift is 0.02 nm. The lattice spacing of d_(002)M1_ and d_(200)R_ is almost the same, 0.227 nm. (b) Structural model of M1 (blue) and T (orange) phases focusing on V atoms shift. Slit angle differences in the structural model are not shown. Compared to the M1 phase, the T phase has a zigzag chain of V atoms every other row. The lattice spacing of d_(020)T_ is 0.243 nm, corresponding to 4.1 nm^−1^.

**Figure 5.**
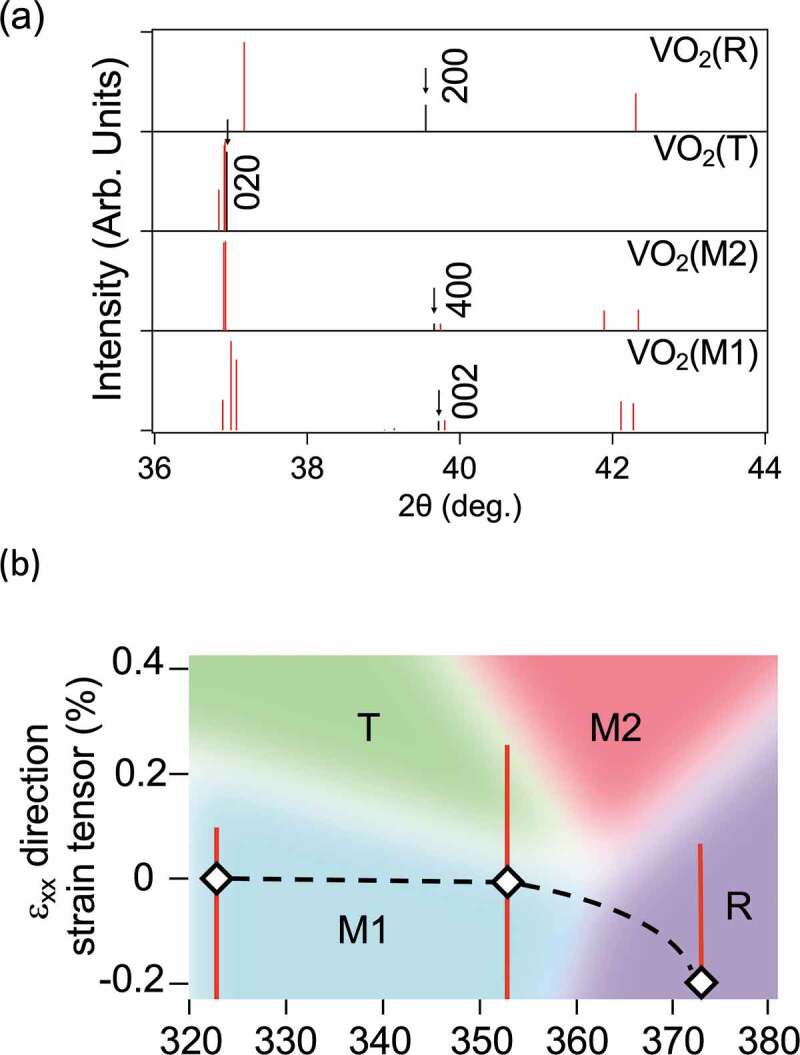
(a) XRD peaks between 2θ = 36 and 44 degrees in JCPDS plots of VO_2_ (M1) and reported previous works of VO_2_(M2) [22], VO_2_(T) [12], and VO_2_(R) [33]. The black lines in stand for Bragg peaks that can be appeared in the spots of Figure 1(c–f). (b) Strain-temperature phase diagram of VO_2_ suggested by D. H. Cobden *et* *al.* (modified) [34]. The diamond marks indicate the averaged strain deduced by GPA. The length of the bars indicates more than 2000 counts shown in Figure 3. The dotted line means an estimated change in average strain during the phase transition process.

Next, to check the strain state at 353 K, GPA was performed for the acquired HRTEM images. The lattice mismatch between the VO_2_ (M1 and R phases) and Al_2_O_3_(0001) substrates is 4.50% and 4.92% parallel to the interface, respectively. Figure 2(a–c) are HRTEM images of the vicinity of the substrate at each temperature. Figure 2(d–i) are GPA images at each temperature and orientation. The inset shows the FFT image of the region surrounded by the white dashed line in Figure 2(a–c). The black dashed line is the boundary of a single crystal domain of VO_2_. The VO_2_[100]_R_ direction along the domain boundary is the x-axis of the strain component, and the VO_2_[001]_R_ direction is the y-axis. Strain components ε_xx_ and ε_yy_ visualize the strain state in the VO_2_ domain. Especially in 353 K the distortion is concentrated in the central domain of the ε_xx_ image, which is only under the compressive strain of below 0.7% but also under the tensile strain of 0.2% (Figure 3). Although the strain is almost zero on average in 353 K, the width of the peak is broader, i.e. the area containing the tensile strain is −0.7 to +0.4%. The ε_yy_ images showed that the strains are concentrated near the interface. The compressive strain applied in the x-direction change significantly at each of the temperatures of 323 K and 373 K. This VO_2_ thin film is formed so that nanorods are growing in the x-direction. As shown in Figure 4(a,b), The change from the M1 phase to the R phase (or vice versa) is accompanied by a volume change that changes the angle and the lattice constant. In the x-direction, there is a space in which the crystal volume change is possible. However, for the y-direction, a phase transition with a large volume change is difficult because another VO_2_ domain exists right next to those. This can result in increased compressive strain with anisotropy at 373 K, as shown in Figure 5(b). As shown in Figure 3, both compressive and tensile strains are present at 323K in the nanoscale range, but they are offset in total. This situation may be the reason for the wide range in strains, although it is plotted where there are no strains in total at 323 K, as shown in Figure 5(b).

To interpret the results of all the above analyses, the atomic arrangement of the T phase was considered together with that of the M1 and R phases. A schematic diagram of V atoms shifts in the M1-R phase transition is illustrated in Figure 4(a). As is well known, the V atoms aligned in the c-axis direction of the rutile-type structure form a zigzag chain at low temperatures, with the V atoms alternately shifting by 0.02 nm. This structural change reduces the symmetry and changes the structure from Tetragonal to Monoclinic. The lattice spacing of d(002)_M1_ and d(200)_R_ is almost equal, 0.227 nm. In contrast, the structural models of the M1 phase (blue) and T phase (orange) focusing on the shift of V atoms are illustrated in Figure 4(b), although the slight difference in angle is not shown. Compared to the M1 phase, the T phase has a zigzag sequence of V atoms every other row. The lattice spacing of d(020)_T_ is 0.243 nm, corresponding to 4.1 nm^−1^ in reciprocal space. The peaks of XRD between 2θ = 36 and 44 degrees in previous works of VO_2_(M2) [22], VO_2_(T) [12], and VO_2_(R) [33] are shown in Figure 5(a) (Full range XRD data are shown in Supporting Information, Figure S5). The black lines in Figure 5(a) stand for Bragg peaks that can be appeared in the TED spots of Figure 1(c–f).

The zigzag chains of the T phase are aligned perpendicular to the [100]_R_ direction every cycle, which can be considered as stacking defects generated during the phase transition from R to M1. The T phase can be mixed with the M1 as a metastable structure. While the angle β of the unit cell in the M1 phase is 122.6 degrees, the corresponding angle in the T phase is angle α, which is 65.0 degrees (supporting information Table S1). Since the obtuse angle of the parallelogram of the unit cell of the M1 phase is wider in the T, this structural change in the T compared to the M1 means that the volume change in the x-axis direction, that is, the direction parallel to the [100]_R_ direction, is more prominent. This difference in the angle of the structural phase is consistent with the fact that the VO_2_ domains at 353 and 373 K are more strained in both parallel and perpendicular directions in the [100]_R_ direction than those in the M1 phase, especially compressive strain at higher temperatures.

The major difference between the characteristics of phase transitions on the nanoscale and those on the microscale is that the amount and type of strain can change during the phase transition process. The modified strain-temperature phase diagram for VO_2_ proposed by D. H. Cobden *et al*. is shown in Figure 5(b) [34]. Since their results were obtained by Raman spectroscopy at the microscale under external stress, the metastable T phase can be observed at the nanoscale even under low strain states. The diamond marks stand for the strain peaks in Figure 3. The bar is in the range of 2000 counts or more in the histogram obtained from our GPA (Figure 3). The dotted line means an estimated change in average strain during the phase transition process. If a region of M2 phase existed in our samples, a diffraction spot of VO_2_(400)_M2_ should appear at 373 K as shown in Figure 5(a). However, the spot did not appear in the actual TED pattern. This suggests a single rutile phase at 373 K. Our measurements suggest that the degree of strain depends on the external field in the structural phase transition process of VO_2_, implying that the control of the single domain or nanostructure is crucial.

Finally, we discuss the possibility of generating a new strain long-period structure as described in the previous paragraph. The strained component appears to have a superstructure with a period of 0.24 nm, corresponding to 4 nm^−1^ in reciprocal space (shown in supporting information, Figure S4). This period is close to the value of *Δq* shown in Figure 1(i). However, if a long-period structure occurred, a peak should also appear near the 000-transparent spot, but no clear peak was observed. Assuming that a long-period strain exists in the x-direction (VO_2_[100]_R_), its half-period is 4 ± 0.5 nm, as shown in Figure S4. In reciprocal space, this value corresponds to 0.222–0.285 nm^−1^. Even if spots due to long-period strain appear, they should be broad, not the sharply peaked spots observed here. Indeed, the corresponding peaks were not identified in the line profiles obtained from the fast Fourier transform (FFT) for the HRTEM image (Figure S3(e)). Therefore, the possibility of a long-period structure of strain was ruled out.

The GPA in this study directly shows that an obstacle in the direction of the volume change associated with the phase transition causes strains in the VO_2_ nanocrystal, which in turn affects the phase transition phase. Due to the strain caused by the lattice mismatch between the sapphire substrate interface and VO_2_, it has been reported that strains are internalized at the interface. The strains act to lower the phase transition temperature of MIT [2]. These reports refer to the structural phase change within only 10 nm from the interface; however, there is no direct indication that strains can occur in the structural phase transition process of the entire domain shown in this study. There was a large difference in the change rate of electrical conductivity near the high-temperature phase and near the low-temperature phase of VO_2_ (Supporting information Figure S1(a)). The mixture of the two phases in the low-temperature phase may cause this difference. Stable and precise control of the phase structure of VO_2_ is essential to fabricate the desired nanostructure. We believe that this study is of great significance as it demonstrates the possibility of controlling the intermediate phase of VO_2_ in the future. Recently, VO_2_ materials have been considered promising candidates for optical switches, which undergo ultrafast light-induced structural phase transitions on the order of   100 femtoseconds [3,4,35–38]. However, little analysis has been done taking strain into account. Our study also suggests that it is meaningful to evaluate the implied strain when tracing the structural changes in VO_2_ induced by ultrafast pulsed light irradiation.

## 4. Conclusion

In conclusion, we have provided crystallographic evidence by electron diffraction that the MIT intermediate phase exists while quantitatively characterizing the strain in VO_2_ thin film grown on sapphire using GPA. The new diffraction spots at 353 K suggested the presence of a phase with the d-spacing of 0.243 nm (4.1 nm^−1^) in the [100]_R_ orientation. The GPA shows the tensile strain of −0.7 to +0.4% (Rutile phase VO_2_[100] direction) in the single domain at 353 K and 373 K compared to that at 323 K, which may cause the T phase to hybridize with the M1 phase. Those methods used in this study could be helpful for precise control of the nanostructure of VO_2_, monitoring the variable strain field during the phase transition process in a nanoscale domain.

## Supplementary Material

Supplemental Material
