# Supplementary material for: Quantitative spatial mapping of distorted state phases during the metal-insulator phase transition for nanoscale VO2 engineering
Source: Sci Technol Adv Mater. 2022 Dec 23;24(1):1–9. doi: 10.1080/14686996.2022.2150525 (PMC9793943; doi:10.1080/14686996.2022.2150525)
Supplement: Supplemental Material [file TSTA_A_2150525_SM8785.docx]

SUPPORTING INFORMATION

Yuichi Ashida ^a^, Takafumi Ishibe ^a^, Jinfeng Yang ^b^, Nobuyasu Naruse ^c^* and Yoshiaki Nakamura ^a^

^a^ Graduate School of Engineering and Science, Osaka University, Toyonaka City, Japan

^b^ The Institute of Scientific and Industrial Research, Osaka University, Ibaraki City, Japan

^c^ Department of Fundamental Bioscience, Shiga University of Medical Science, Otsu City, Japan

*Corresponding author

E-mail address: naruse@belle.shiga-med.ac.jp

Table S1. Lattice parameters of carious phase VO_2_ were taken from previous studies [1,2]. Lattice parameters of various phase VO_2_ were taken from previous studies [1,2]. The axial orientation of phase T phase is roughly parallel to a_T_ and b_M1_, b_T_ and c_M1_, and c_T_ and a_M1_. There is the following correspondence between the angles of phase T and M1 phase. α_T_ = 65.0º and β_M1_ = 122.6º, β_T_ = 88.83º and α_M1_ = 90.0º, γ_T_ = 88.8º and γ_M1_ = 90.0º.

| Crystal name | Space group | Structure | a (Å) | b (Å) | c (Å) | β (deg.) |
| --- | --- | --- | --- | --- | --- | --- |
| VO_2_(M_1_) | P2_1_/c | Monoclinic | 5.753 | 4.526 | 5.383 | 122.6 |
| VO_2_(M_2_) | C2/m | Monoclinic | 9.083 | 5.763 | 4.532 | 91.30 |
| VO_2_(T) | P$\bar{1}$ | Triclinic | 4.523 | 5.364 | 5.378 | --- |
| VO_2_(R) | P4_2_/mnm | Tetragonal | 4.555 | 4.555 | 2.856 | 90.00 |

Figure 1S. (a) The electric conductivity (σ) values in VO_2_ thin film on Al_2_O_3_ substrate in the in-plane direction were measured by the van der Pauw method. (b) XRD measurements were carried out using the Cu K_α_ and K_β_ line (wavelength, 0.15418 and 0.13847 nm).


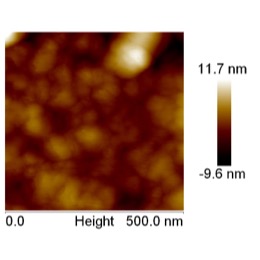


Figure 2S. AFM Image of VO_2_ thin film surface. Confirmed average domain size equal to ~20 nm by line profiles.


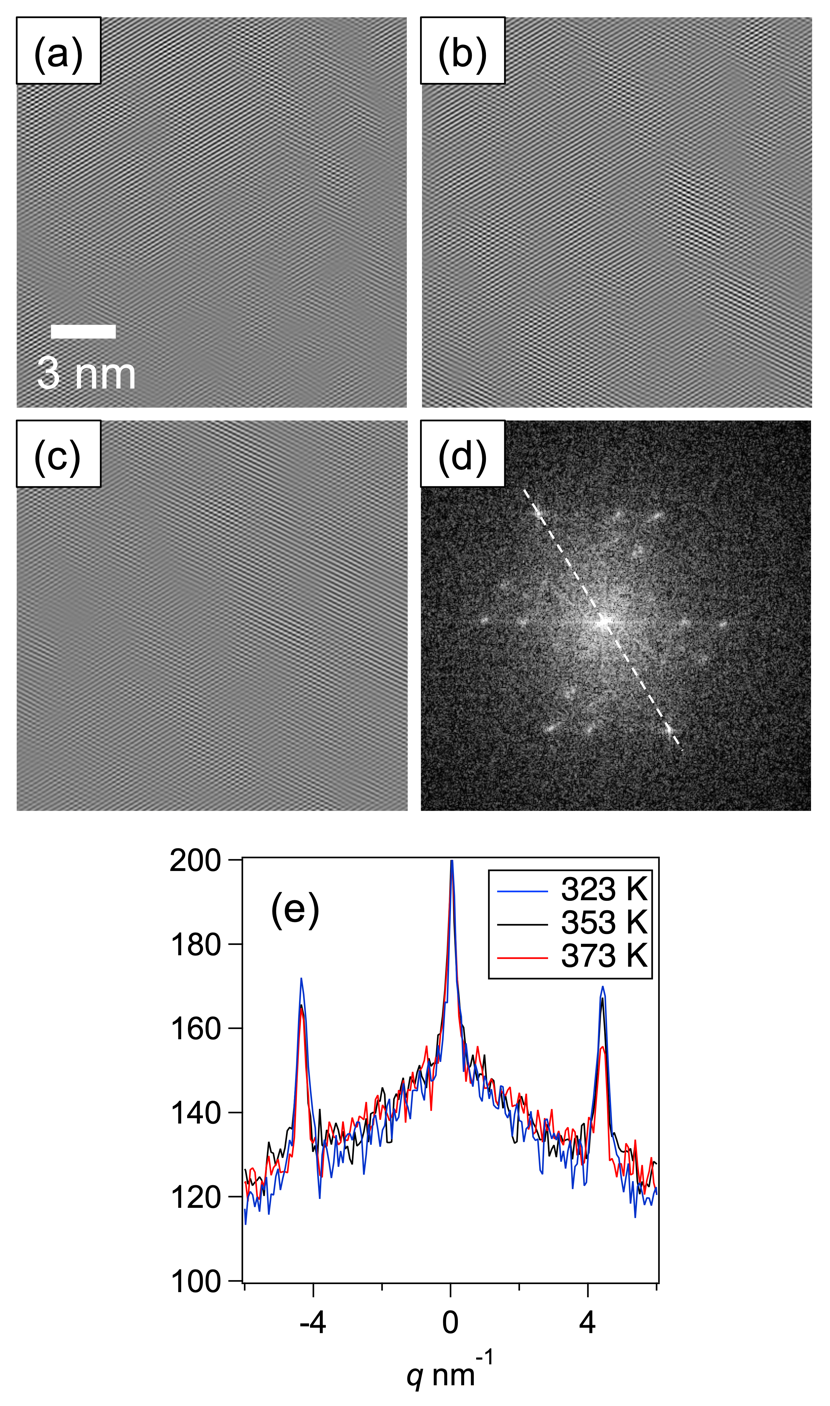


Figure 3S. Magnified image (HRTEM) of the white dotted line region in Figures 2(a-c). (a) 373 K, (b) 353 K, and (c) 323 K. (d) FFT image of the region in (a). (e) Line profiles of FFT pattern (Inset the Fig. 2(a-c)) same direction of Figure 1(a) white dotted line. The red, black, and blue lines indicate 373 K, 353 K, and 323 K, respectively; no new peaks appeared due to temperature change.

Figure 4S. Line profile for the sandwiched domain in Figure 2(e) along the VO_2_[100]_R_ direction. Since the GPA image in Figure 2(e) is inherently grayscale, the vertical axis represents its brightness.

Figure 5S. X-ray diffraction (XRD) pattern in JCPDS plots of VO_2_(M1) and reported previous works of VO_2_(M2) [1], VO_2_(T) and VO_2_(R) [2].

Reference

[1] Chamberland B L 1973 New Defect Vanadium Dioxide Phases *JOURNAL OF SOLID STATE CHEMISTRY* **7** 377–84.

[2] Ghedira M, Vincent H, Marezio M and Launay J C 1977 Structural Aspects of the Metal-Insulator Transitions in V_0.985_Al_0.015_O_2_ *JOURNAL OF SOLID STATE CHEMISTRY* **22** 423–38.
